# Predictors of adherence to public health behaviors for fighting COVID-19 derived from longitudinal data

**DOI:** 10.1038/s41598-021-04703-9

**Published:** 2022-03-09

**Authors:** Birga M. Schumpe, Caspar J. Van Lissa, Jocelyn J. Bélanger, Kai Ruggeri, Jochen Mierau, Claudia F. Nisa, Erica Molinario, Michele J. Gelfand, Wolfgang Stroebe, Maximilian Agostini, Ben Gützkow, Bertus F. Jeronimus, Jannis Kreienkamp, Maja Kutlaca, Edward P. Lemay, Anne Margit Reitsema, Michelle R. vanDellen, Georgios Abakoumkin, Jamilah Hanum Abdul Khaiyom, Vjollca Ahmedi, Handan Akkas, Carlos A. Almenara, Mohsin Atta, Sabahat Cigdem Bagci, Sima Basel, Edona Berisha Kida, Allan B. I. Bernardo, Nicholas R. Buttrick, Phatthanakit Chobthamkit, Hoon-Seok Choi, Mioara Cristea, Sara Csaba, Kaja Damnjanović, Ivan Danyliuk, Arobindu Dash, Daniela Di Santo, Karen M. Douglas, Violeta Enea, Daiane Faller, Gavan J. Fitzsimons, Alexandra Gheorghiu, Ángel Gómez, Ali Hamaidia, Qing Han, Mai Helmy, Joevarian Hudiyana, Ding-Yu Jiang, Veljko Jovanović, Zeljka Kamenov, Anna Kende, Shian-Ling Keng, Tra Thi Thanh Kieu, Yasin Koc, Kamila Kovyazina, Inna Kozytska, Joshua Krause, Arie W. Kruglanski, Anton Kurapov, Nóra Anna Lantos, Cokorda Bagus J. Lesmana, Winnifred R. Louis, Adrian Lueders, Najma Iqbal Malik, Anton P. Martinez, Kira O. McCabe, Jasmina Mehulić, Mirra Noor Milla, Idris Mohammed, Manuel Moyano, Hayat Muhammad, Silvana Mula, Hamdi Muluk, Solomiia Myroniuk, Reza Najafi, Boglárka Nyúl, Paul A. O’Keefe, Jose Javier Olivas Osuna, Evgeny N. Osin, Joonha Park, Gennaro Pica, Antonio Pierro, Jonas H. Rees, Elena Resta, Marika Rullo, Michelle K. Ryan, Adil Samekin, Pekka Santtila, Edyta Sasin, Heyla A. Selim, Michael Vicente Stanton, Samiah Sultana, Robbie M. Sutton, Eleftheria Tseliou, Akira Utsugi, Jolien A. van Breen, Kees Van Veen, Alexandra Vázquez, Robin Wollast, Victoria Wai-Lan Yeung, Somayeh Zand, Iris Lav Žeželj, Bang Zheng, Andreas Zick, Claudia Zúñiga, N. Pontus Leander

**Affiliations:** 1 University of Amsterdam, Amsterdam, The Netherlands; 2 Utrecht University, Utrecht, Netherlands; 3 New York University Abu Dhabi, Abu Dhabi, United Arab Emirates; 4 Columbia University, New York, USA; 5 University of Groningen, Groningen, Netherlands; 6 Florida Gulf Coast University, Fort Myers, USA; 7 Stanford University, Palo Alto, USA; 8 Durham University, Durham, UK; 9 University of Maryland, College Park, USA; 10 University of Georgia, Athens, USA; 11 University of Thessaly, Volos, Greece; 12 International Islamic University Malaysia, Kuala Lumpur, Malaysia; 13 Pristine University, Pristine, Kosovo; 14 Ankara Science University, Ankara, Turkey; 15 Universidad Peruana de Ciencias Aplicadas, Lima, Peru; 16 University of Sargodha, Sargodha, Pakistan; 17 Sabanci University, Istanbul, Turkey; 18 De La Salle University, Manila, Philippines; 19 University of Virginia, Charlottesville, USA; 20 Thammasat University, Pathumthani, Thailand; 21 Sungkyunkwan University, Seoul, Korea; 22 Heriot Watt University, Edinburgh, UK; 23 ELTE Eötvös Loránd University, Budapest, Hungary; 24 University of Belgrade, Belgarde, Serbia; 25 Taras Shevchenko National University of Kyiv, Kyiv, Ukraine; 26 Leuphana University of Lüneburg, Lüneburg, Germany; 27 University “La Sapienza”, Rome, Italy; 28 University of Kent, Canterbury, UK; 29 Alexandru Ioan Cuza University, Iași, Romania; 30 National University of Singapore, Singapore, Singapore; 31 Duke University, Durham, USA; 32 Universidad Nacional de Educación a Distancia, Madrid, Spain; 33 Setif 2 University, Setif, Algeria; 34 University of Bristol, Bristol, UK; 35 Sultan Qaboos University, Muscat, Oman; 36 Universitas Indonesia, Depok, Indonesia; 37 National Chung-Cheng University, Minxiong, Taiwan; 38 University of Novi Sad, Novi Sad, Serbia; 39 University of Zagreb, Zagreb, Croatia; 40 Monash University Malaysia, Subang Jaya, Malaysia; 41 HCMC University of Education, Ho Chi Minh City, Vietnam; 42 Nur-Sultan, Kazakhstan; 43 University of Groningen, Kazakhstan, Netherlands; 44 Udayana University, Denpasar, Indonesia; 45 University of Queensland, Brisbane, Australia; 46 University of Limerick, Limerick, Ireland; 47 University of Sheffield, Sheffield, UK; 48 Carleton University, Ottawa, Canada; 49 Usmanu Danfodiyo University Sokoto, Sokoto, Nigeria; 50 University of Cordoba, Cordoba, Spain; 51 University of Peshawar, Peshawar, Pakistan; 52 University of Padova, Padova, Italy; 53 Yale-NUS College, Singapore, Singapore; 54 National Distance Education University (UNED), Madrid, Spain; 55 HSE University, Moscow, Russia; 56 NUCB Business School, Nagoya, Japan; 57 University of Camerino, Camerino, MC Italy; 58 University of Bielefeld, Bielefeld, Germany; 59 University of Siena, Siena, Italy; 60 University of Exeter, Exeter, UK; 61 M. Narikbayev KAZGUU University, Nur-Sultan, Kazakhstan; 62 New York University Shanghai, Shanghai, China; 63 King Saud University, Riyadh, Saudi Arabia; 64 California State University, East Bay, USA; 65 Nagoya University, Nagoya, Japan; 66 Leiden University, Leiden, Netherlands; 67 Université Clermont-Auvergne, Clermont-Ferrand, France; 68 Lingnan University, Tuen Mun, Hong Kong; 69 University of Milano-Bicocca, Milan, Italy; 70 Imperial College London, London, UK; 71 Bielefeld University, Bielefeld, Germany; 72 Universidad de Chile, Santiago, Chile; 73 Wayne State University, Detroit, USA

**Keywords:** Human behaviour, Health policy, Lifestyle modification, Preventive medicine, Epidemiology, Population screening

## Abstract

The present paper examines longitudinally how subjective perceptions about COVID-19, one’s community, and the government predict adherence to public health measures to reduce the spread of the virus. Using an international survey (*N* = 3040), we test how infection risk perception, trust in the governmental response and communications about COVID-19, conspiracy beliefs, social norms on distancing, tightness of culture, and community punishment predict various containment-related attitudes and behavior. Autoregressive analyses indicate that, at the personal level, personal hygiene behavior was predicted by personal infection risk perception. At social level, social distancing behaviors such as abstaining from face-to-face contact were predicted by perceived social norms. Support for behavioral mandates was predicted by confidence in the government and cultural tightness, whereas support for anti-lockdown protests was predicted by (lower) perceived clarity of communication about the virus. Results are discussed in light of policy implications and creating effective interventions.

## Introduction

On March 11th, 2020, the World Health Organization (WHO) declared COVID-19 a pandemic. Since then, the novel coronavirus SARS-CoV-2, which leads to the illness referred to as COVID-19, has put society to the test. From washing hands to getting vaccinated, individual health behavior is the most important defense to curb the spread of the virus ^1,2^, behavioral sciences need to be leveraged to build trust that encourages individuals to fully utilize public health recommendations ^3^.

## Predicting adherence to public health measures

Based on existing evidence regarding health behaviors, we focus this work on personal risk perception (first-order effects), subjective social and community norms (second-order effects), and broader political and governmental perceptions (third-order effects).

### First-order effects

Individuals’ perceived personal risk of infection is often studied in the context of communicable diseases such as H1N1 (‘Swine Flu’) or the original SARS outbreaks in 2002–2003. A greater perception of personal risk was strongly related to increased willingness to take precautionary measures against infection ^4–6^. Protection Motivation Theory ^7^ proposes that the severity of a threatening event and the perceived probability of its occurrence determines whether people engage in healthy behaviors. Recent findings indicate that perceived economic risk is also positively related to mitigation behavior and policy support ^8^.

Given these insights, we hypothesize that personal risk perception regarding COVID-19 predicts willingness to engage in protective health behaviors and support for pandemic response related policies. Importantly, this hypothesis has not previously been tested longitudinally, hence, claims of causality are not possible.

### Second-order effects

Social psychology has a long history of observing that *social norms—*the subjective perception of what others are doing and approving of—are a reliable predictor for people’s behavior across contexts ^9–11^. Likewise, social norms play an important role in predicting health behaviors ^12,13^. Specifically, for the COVID-19 pandemic, social distancing norms might predict compliance with health measures. We predict that injunctive social norms, that is, norms of “ought” ^14^ would predict more compliance over time with public health measures.

Injunctive norms may be manifested in what community perceive they and others ought to do as well as through subjective perceptions that norm violators are punished. That is, people may perceive their community would impose *punishments* (e.g., scorn or fines) for not following public health recommendations enforced by government authorities. Examples of these behaviors include breaking quarantine or not wearing face masks.

But does the perception of a punitive community encourage compliance in individuals? On the one hand, increasing the intensity or duration of punishments can lead to greater suppression of targeted (unwanted) behaviors ^15^. However, individuals are more likely to resist when they perceive their freedom to engage in a specific behavior is threatened ^16^, especially if they perceive such resistance as the norm among their ingroup ^17^. Thus, we test the predictive power of perceived punishments for adherence to healthy behavior recommendations from public health officials over time.

### Third-order effects

On a general level, trust in the government is a predictor for adherence to recommended health behaviors ^18,19^. In contrast, distrust in the government is known to be related to lower compliance with, for example, policies aimed at stemming the Ebola outbreak ^20^. In the COVID-19 context, cross-sectional studies support this assertion: people in the US who fear the authorities comply less with mitigation measures to fight COVID-19 ^21^. We predict that, over time, trust in the government to effectively fight COVID-19 would lead to greater compliance with public health recommendations and greater support for governmental policies to mitigate the COVID-19 pandemic.

Further, how risks are communicated is a critical consideration ^22^. Subjective perceptions that one receives *clear and unambiguous messages* are deemed central for promoting compliance with public health measures ^23^. Taken together, we expect that individuals who perceive to receive clear and unambiguous messages would show increased adherence to public health recommendations over time.

In contrast to earlier points, the pandemic has fueled numerous misperceptions about the virus and society, including *conspiracy theories*. For example, some people believe that the coronavirus was created to be used as a population-control scheme. False beliefs regarding vaccines (e.g., that they contain chips or lead to genetic modification) can result in deaths if individuals fail to get protected and avoidable transmission occurs. Indeed, belief in conspiracy theories predicts resistance to preventive behaviors ^24^.

People may also perceive that society ought to tighten or loosen its injunctive norms. An important societal level factor in cross-cultural research is *tightness-looseness* ^24^. Tightness is defined by having strong norms, strict rules, and low tolerance of deviating behavior, often considered characteristic of places such as Singapore or Japan. Loose cultures, such as Italy or the United States, are characterized by weaker social norms and rules and being generally more permissive about deviation ^25^. Since people in tighter cultures tend to follow rules more strictly and are more accepting toward authoritarian leadership, cultural tightness should predict compliance with public health measures. Importantly, a stronger preference for tighter structures after the onset of the pandemic would be indicative of greater perception that societal circumstances demand for more control and rule enforcement during these times ^26,27^.

In sum, prior research offers some evidence that subjective perceptions at the personal-, social-, or societal level may predict various pandemic-related attitudes and behaviors. Yet, the existing literature has several limitations. Firstly, no study has comprehensively investigated predictors at all three levels of analysis (personal-, social-, and societal level). However, to design effective interventions, it would be useful to know whether certain subjective perceptions are generally predictive, or whether a given subjective perception is most predictive of a relevant outcome when considered at the same level of construal—be it at the personal-level, the social, or a more generalized societal level ^28^. Such an analysis can also offer preliminary indication of whether and how future research and policy should tailor interventions on subjective perception to the specific outcome of interest.

Second, most studies have examined these factors in the context of infectious diseases other than COVID-19 ^29^. The COVID-19 pandemic required instantaneous behavioral change at a global level. This mass nature of the COVID-19 pandemic requires an understanding that cannot necessarily be translated from other infectious diseases. For instance, the novel coronavirus (2019-nCoV) has a higher transmissibility than SARS (SARS-CoV) and more patients with mild symptoms that fail to be isolated ^2^. As this occurred in a period when global travel was much more accessible than in 2002, it meant a potentially large number of individual carriers were potentially unknowingly spreading the disease before public health officials had a chance to react.

Lastly, the studies that did address COVID-19 have been primarily of cross-sectional nature only ^6,18^. This means previous research has not provided any information on temporal precedence of the effects. For example, we do not know whether those subjective perceptions are indeed antecedents even though social psychology has long recognized that subjective perceptions can change to match their prior behavior (e.g., self-perception theory) ^30^. The present research seeks to overcome these limitations by studying predictors at all three levels of analysis over time in the context of COVID-19 to establish temporal precedence. In doing so, we aim to identify factors that predict compliance with preventative interventions that would allow policy makers to craft effective interventions to mitigate the COVID-19 pandemic through behaviors such as hand washing, quarantining, and social distancing.

## Results

We tested whether the hypothesized predictors were reliably associated with changes in health behavior and support for public health recommendations, while controlling for stability of the dependent variable and the influence of age, gender, employment status, education, religion, political view, date survey taken, time interval between measurements, as well as subjective proximity to COVID-19 cases.

We standardized all predictors with respect to the sample mean and standard deviation. This was considered optimal as standardized coefficients are not currently available for multilevel models with random slopes estimated in Mplus. Such grand-mean centering is typically used when within-cluster effects are not the primary target of inference ^31^. The regression coefficients of the standardized predictors can be interpreted as approximate indicators of relative variable importance, albeit disregarding differences across levels and random effects (i.e., differences in variable importance across countries). For each hypothesized effect, we report whether the average effect was significant across countries and whether there was significant between-country variance in the effect. The multilevel design was hence employed to address the country-level hypotheses.

### Adherence to recommended health behaviors

Table 1 shows the theoretically-derived variables that reliably predicted adherence to health behaviors recommended by public health guidelines. With regards to personal hygiene, hand washing (LL = −3950.25, AIC = 7984.50, BIC = 8241.23) showed a significant autoregressive effect (*B* = 0.80; *p* < 0.001; CI [0.54, 1.06]), indicating stability across the waves. This stability varied significantly across countries (*B* = 0.19; *p* < 0.001; CI [0.07, 0.31]). After controlling for this autoregressive effect, changes in hand washing were predicted by perceived risk to becoming infected (*B* = 0.05; *p* < 0.001; CI [0.02, 0.09]), belief in conspiracy theories (*B* = 0.04; *p* < 0.001; CI [0.02, 0.07]), and perceptions that one receives clear and unambiguous messages about what to do about the coronavirus (*B* = 0.04; *p* > 0.001; CI [0.01, 0.08]). This suggests that, over time, individuals who perceive greater risk to becoming infected, greater conspiracies, and greater message clarity washed their hands more frequently. These effects did not differ between countries, *ps* > 0.40.

**Table 1:** Predictors of health behaviors.

Health behavior	Predictor	*B*	*p*	CI
Hand washing	Perceived risk to becoming infected	0.05	< 0.001	[0.02, 0.09]
	Belief in conspiracy theories	0.04	< 0.001	[0.02, 0.07]
	Getting clear and unambiguous messages about what to do	0.04	< 0.001	[0.01, 0.08]
Avoiding crowds	Social norms	0.16	< 0.001	[0.09, 0.23]
	Perceived risk to becoming infected	0.06	< 0.001	[0.03, 0.10]
	Preference for cultural tightness	0.04	< 0.001	[0.02, 0.06]
	Belief in conspiracy theories	0.02	0.01	[0.01, 0.04]
Self-isolation/quarantine	Social norms	0.09	0.05	[0.00, 0.18]
Face-to-face contact with friends and family	Social norms	−0.17	< 0.001	[−.24, −0.09]
Face to face contact with other people	Social norms	−0.14	< 0.001	[−0.22, −0.07]
	Preference for cultural tightness	−0.06	0.04	[−0.11, −0.00]
Days per week people left their house	Social norms	−0.06	< 0.001	[−0.08, −0.04]
	Cultural tightness	−0.04	0.04	[−0.07, −0.00]

Willingness to wear a face mask (LL = -1806.83, AIC = 3669.67, BIC = 3809.73) showed high stability across waves (*B* = 2.02; *p* < 0.001; CI [1.38, 2.21]). This stability did not vary between countries, *p* > 0.79. When controlling for the autoregressive effect, changes in willingness to wear a face mask were predicted by subjective proximity to COVID-19 cases (*B* = 0.30; *p* = 0.05; CI [0.00, 0.59]). This effect did not differ between countries, *p* > 0.99.

Avoiding crowds (LL = −3719.47, AIC = 7522.94, BIC = 7779.68) showed a significant autoregressive effect (*B* = 0.59; *p* < 0.001; CI [0.44, 0.73]). This stability varied significantly across countries, *B* = 0.17; *p* < 0.001; CI [0.09, 0.25]). Changes in avoiding crowds were significantly predicted by social distancing norms (*B* = 0.16; *p* < 0.001; CI [0.09, 0.23]), risk perception to becoming infected (*B* = 0.06; *p* < 0.001; CI [0.03, 0.10]), people’s preference for tight cultural structures (*B* = 0.04; *p* < 0.001; CI [0.02, 0.06]), and their belief in conspiracy theories (*B* = 0.02; *p* = 0.01; CI [0.01, 0.04]). Hence, stronger norms regarding social distancing, higher perceived risk to becoming infected, and a stronger preference for tight cultural structures predicted over-time increase in the tendency to avoid crowds. Only the effect for social norms varied across countries (*B* = 0.06; *p* = 0.01; CI [0.02, 0.10]). Further, authoritarianism (*B* = 0.04; *p* < 0.001; CI [0.01, 0.06]) and participants’ age (*B* = 0.05; *p* = 0.01; CI [0.01, 0.08]) positively predicted change over time in people’s tendency to avoid crowds. This suggests that, over time, older and more authoritarian participants avoided crowds more. These effects did not vary significantly across countries, *ps* > 0.82. Gender (*B* = −0.05; *p* = 0.04; CI [0.10, −0.00] was a significant predictor, with women showing greater change in the tendency to avoid crowds. Date of survey participation was also predictive; participants who enrolled later showed decreases in the tendency to avoid crowds over-time (*B* = −0.01; *p* = 0.01; CI [−0.02, −0.01]). These effects did not differ between countries, *p*s > 0.09.

Quarantining (LL = −5320.38, AIC = 10,724.75, BIC = 10,981.48) showed a significant autoregressive effect (*B* = 1.06; *p* < 0.001; CI [0.96, 1.17]), which varied across countries (*B* = 0.28; *p* < 0.001; CI [0.20, 0.35]). Changes in quarantining were predicted by social distancing norms (*B* = 0.09; *p* = 0.05; CI [0.00, 0.18]), time between measurements (*B* = −0.13; *p* < 0.001; CI [−0.20, −0.06]), employment status (*B* = −0.09; *p* = 0.01; CI [−0.16, −0.03]), age (*B* = −0.07; *p* < 0.001; CI [−0.11, −0.04]), and the date the survey was taken (*B* = −0.02; *p* < 0.001; CI [−0.03, −0.01]). None of these effects did vary significantly across countries, *p*s > 0.05.

In-person (face-to-face) contact with friends and family (LL = −7422.56, AIC = 14,929.13, BIC = 15,190.06) showed significant stability over time (*B* = 1.04; *p* < 0.001; CI [0.92, 1.17]), which varied across countries (*B* = 0.75; *p* < 0.001; CI [0.60, 0.90]). Changes in social face-to-face contact with friends and family were predicted by social norms on distancing (*B* = −0.17; *p* < 0.001; CI [−0.24, −0.09]), participants’ age (*B* = −0.19; *p* < 0.001; CI [−0.28, −0.09]) and gender (*B* = 0.16; *p* = 0.02; CI [0.03, 0.29]). Overall, social distancing norms were the best predictor for changes in face-to-face contact. Older participants and men had less in-person contact with friends and family. These effects did not differ between countries, *p*s > 0.24.

Social face to face contact with other people in general (“others”, LL = −7272.44, AIC = 14,628.88, BIC = 14,889.61) showed significant stability over time (*B* = 1.12; *p* < 0.001; CI [1.04, 1.21]). This autoregressive effect varied across countries (*B* = 0.45; *p* < 0.001; CI [0.34, 0.57]). Controlling for this, changes in social face to face contact with others were predicted by social distancing norms (*B* = −0.14; *p* < 0.001; CI [−0.22, −0.07]) and preference for tightness (*B* = −0.06; *p* = 0.04; CI [−0.11, −0.00]. Another predictor was employment status (*B* = 0.48; *p* < 0.001; CI [0.29, 0.66]), which differed across countries (*B* = 0.85; *p* > 0.001; CI [0.39, 1.31]), all other *p*s > 0.10.

The number of days in a week people left their house (LL = −3861.18, AIC = 7806.35, BIC = 8067.47) showed significant stability over time (*B* = 0.79; *p* < 0.001; CI [0.76, 0.82]). Changes in the number of days in a week people leaving their house were predicted by social norms on distancing (*B* = −0.06; *p* < 0.001; CI [−0.08, −0.04]), cultural tightness (*B* = −0.04; *p* = 0.04; CI [−0.07, −0.00]), being employed (*B* = 0.13; *p* < 0.001; CI [0.08, 0.18]), and being politically on the right side of the spectrum (*B* = 0.02; *p* = 0.04; CI [0.00, 0.04]). There were no between country differences in these effects, all *p*s > 0.26.

### Attitudes toward behavioral mandates

Table 2 shows the theoretically derived variables that predict attitudes toward behavioral mandates. People’s support for mandatory vaccination (LL = −5688.01, AIC = 11,460.03, BIC = 11,719.13) showed significant stability over time (*B* = 1.36; *p* < 0.001; CI [1.25, 1.47]). This autoregressive effect varied across countries (*B* = 0.13; *p* = 0.02; CI [0.02, 0.24]). Controlling for it, changes in support for mandatory vaccination were predicted by participants’ trust in the government to fight COVID-19 (*B* = 0.07; *p* = 0.01; CI [0.01, 0.13]). This shows that people support mandatory vaccination more when they have greater trust in the government to fight COVID-19 effectively. Moreover, changes in support for mandatory vaccination were predicted by social distancing norms (*B* = 0.07; *p* = 0.01; CI [0.02, 0.13]), being politically on the right side of the spectrum (*B* = −0.04; *p* = 0.03; CI [−0.08, −0.00]), and the date the survey was taken (*B* = −0.02; *p* < 0.001; CI [−0.03, −0.01]; no between country differences, all *p*s > 0.58).

**Table 2:** Predictors of attitudes toward behavioral mandates.

Attitudes toward mandates	Predictor	*B*	*p*	CI
Mandatory vaccination	Trust in the government to fight COVID-19	0.07	0.01	[0.01, 0.13]
	Social norms	0.07	0.01	[0.02, 0.13]
*Mandatory quarantine*	Social norms	0.17	< 0.001	[0.09, 0.24]
	Trust in the government to fight COVID-19	0.08	< 0.001	[0.02, 0.34]
	Preference for cultural tightness	0.08	< 0.001	[0.02, 0.13]
*Protest containment measures*	Getting clear and unambiguous messages about what to do	−0.07	0.04	[−0.13, −0.00]

Results further revealed that support for mandatory quarantine (LL = −4965.04, AIC = 10,014.08, BIC = 10,273.18) showed significant stability over time (*B* = 0.66; *p* < 0.001; CI [0.58, 0.74]). This autoregressive effect varied significantly across countries (*B* = 0.19; *p* > 0.001; CI [0.12, 0.26]). Controlling for it, individuals’ changes in support for mandatory quarantine were predicted by social distancing norms (*B* = 0.17; *p* < 0.001; CI [0.09, 0.24]), trust in the government to fight COVID-19 (*B* = 0.08; *p* > 0.001; CI [0.02, 0.34]), preference for cultural tightness (*B* = 0.08; *p* < 0.001; CI [0.02, 0.13]), being female (*B* = −0.08; *p* = 0.05; CI [−0.17, −0.00]), age (*B* = 0.05; *p* = 0.01; CI [0.01, 0.10]), authoritarianism (*B* = 0.05; *p* = 0.02; CI [0.01, 0.09]), time interval between initial and follow-up measurement (*B* = −0.04; *p* = 0.01; CI [−0.07, −0.01]), and the date the survey was taken (*B* = −0.01; *p* < 0.001; CI [−0.02, −0.00]). There were no between country differences, all *p*s > 0.36.

People’s readiness to protest containment measures (LL = −1803.26, AIC = 3662.53, BIC = 3806.61) showed a significant autoregressive effect, indicating stability across the waves (*B* = 1.06; *p* > 0.001; CI [0.88, 1.25]). This stability varied significantly across countries (*B* = 0.33; *p* > 0.001; CI [0.19, 0.47]). Controlling for the autoregressive effect, changes in the readiness to protest containment measures were predicted by participants’ perception that they were getting clear and unambiguous messages about the coronavirus (*B* = −0.07; *p* = 0.04; CI [−0.13, −0.00]). Lastly, changes in readiness to protest containment measures was predicted by whether participants were religious or not (*B* = 0.15; *p* > 0.001; CI [0.07, 0.23]; no between country differences, *p*s > 0.09).

## Discussion

For behavioral science to successfully inform public health policy towards mitigating the pandemic, there is a need to conduct holistic, cross-cultural, longitudinal research that identifies the unique effects of various candidate predictors on relevant outcomes of interests. Simultaneously testing multiple candidate predictors can help to pinpoint the most important predictors for a given outcome or a set of outcomes. The global scale of a pandemic may call for a unified global response, which means that a candidate predictor should be tested across cultures to determine its generalizability. A longitudinal approach helps to establish temporal precedence between predictors and outcomes, which gives early insight into potential causal inferences that can be tested with intervention studies.

Given these aims, the present research used a longitudinal design to identify the subjective perceptions that predict individuals’ changes, over time, in adherence to behaviors recommended by public health guidance and support of general public health policies in the context of COVID-19. We specifically tested several predictors that have been found potentially relevant by prior literature, that is, risk perception, social norms, punishments, trust in the government, clear and unambiguous messages, cultural tightness, and belief in conspiracy theories.

Several attitudes and behaviors related to public health (e.g., quarantining, wearing a face mask, etc.) were assessed as critical outcome variables at numerous points in time. This approach helps to determine whether certain factors are generally predictive, across multiple outcomes of interest, or whether different subjective perceptions predict changes in specific virus prevention behaviors and attitudes over time. In this vein, the most generalizable predictor was social and community-level norms, which reliably predicted outcomes across conceptual levels, such as personal hygiene, public contact, and attitudes towards behavioral mandates. In practices, this means public health officials should strongly consider existing and developing social norms not only in what measures are needed to mitigate a pandemic, but also precisely how to communicate those messages. However, social norms did not predict all outcomes and, ultimately, each outcome had its own idiosyncratic set of predictors.

First-order perceptions of perceived individual risk of becoming infected predicted changes in adherence to important health behaviors such as frequent hand washing. A possible implication of these findings could be to install nudges (small changes in choice architecture that encourage optimal decisions without force or genuine changes in the circumstance ^32^) in public bathrooms that are related to concepts of risk infection. At a minimum, our findings suggest a potential utility for nudges that would increase the salience regarding the risk of becoming infected (e.g., pictures of hands in which germs made visible through U.V. light could trigger disgust ^33^.

None of the theoretically-relevant variables predicted a change in willingness to engage in another health behavior. However, particularly for wearing face masks, participants in proximity to infected individuals showed an increased tendency to use face masks. This proximity and familiarity effect may have been even stronger when people know someone in their social network who is infected. Indeed, people make judgments about the frequency or likelihood of events based on how available information is to them ^34^. Given this proximity effect, there is reason for policymakers to consider encouraging individuals to share positive diagnoses, or at least aim to reduce stigma about positive results. If the effect is accurate, being made aware of proximal infections could directly reduce negative attitudes toward healthy behaviors, if not directly increase likelihood of engaging in those behaviors recommended by public health officials. Such a finding can therefore directly inform public health messaging.

Second-order social distancing norms predict changes in social distancing behavior. Public health messages could aim at changing social norms or making existing ones more salient. For instance, common approaches to modifying social norms are to change possible misperceptions of social norms ^35^ or to make certain group memberships more salient ^36^.

Testing third-order effects showed clearly that trust in the government to effectively fight the COVID-19 pandemic predicts increased support for mandatory measures. In other words, as may seem obvious but is critical to state outright: where there is trust in the government to be effective, there is substantially greater support for initiatives to combat a pandemic. Some considerations of this finding mean that, for instance, it is important for governments to have frequent and informative briefings, in which leaders address the nation and give clear instructions on what to do. To build trust from these, there would ideally be information that shows how past guidelines have been effective, and what to expect out of the newest recommendations.

Also, preference for cultural tightness predicted over-time increases in the tendency to support behavioral mandates. This backs the idea that countries with tighter cultures are better prepared to handle the outbreak ^37^. Hence, policy makers should sensitize the public for a temporary need for tighter structures.

To conclude, the present findings specify the subjective perceptions that policy makers should use as levers of intervention for containment of the coronavirus pandemic. Despite the surge in behavioral research related directly to the COVID-19 pandemic, longitudinal analyses are still lacking. Compared to cross-sectional studies, autoregressive longitudinal research can make a stronger claim to causality, because it meets the requirements of Granger causality ^38^.

Despite several strengths, this study also has some limitations. Like most social scientific research, our approach assumed linearity of associations between variables. Unfortunately, this assumption is difficult to check in the structural equation modeling context—but no strong theoretical evidence exists to presume a different shape for the associations presented here. Furthermore, the predictors were chosen based on their theoretical merit. This is not to say that there are no other potential determining factors for the behaviors investigated here such as cultural ones. Although our data comprised multiple countries, we did not consider the potential influence of cultural moderators. It should be noted, however, that 18 countries is a very small sample size for detecting between-country differences. Moreover, almost all random slopes in the study were non-significant, indicating that there was little to no variance to be explained at the between-country level.

The current research can directly inform official public health messaging and intervention efforts. First, the potential effectiveness for nudges to encourage hand washing is appropriate for field-testing. Concurrently, policymakers may wish to focus efforts on raising awareness of proximal infections, which appears to increase the likelihood of engaging in healthy behaviors. In doing so, public health messages should either aim at promoting positive social norms or making existing ones more salient. Finally, transparency in the process for all of these has clear importance and governments should be applied this to maximize effectiveness and build trust for the well-being of the communities they serve.

## Methods

### Participants and design

Our international survey (https://www.psycorona.org) conducted in April 2020 served as the baseline. Participants could opt-in for weekly follow-ups lasting until June 2020. This longitudinal study is the focus of the present analysis. Tables 3 and 4 describe the participants per wave and country in our final analysis (*N* = 3040). Full methodological details, including exact dates of the measurements and description of items/questionnaires in all languages, are provided in the survey codebook (https://osf.io/qhyue). Informed consent was obtained and all methods were performed in accordance with the relevant guidelines and regulations.

**Table 3:** Observations per country for health behaviors and attitudes toward behavioral mandates.

Country	Health behaviors							Attitudes toward behavioral mandates		
	Hand washing	Avoiding crowds	Self-isolation/quarantine	Wearing face mask	Face-to-face contact friends and family	Face to face contact other people	Days per week house left	Mandatory vaccination	Mandatory quarantine	Protest containment measures
USA	94	94	94	34	109	109	109	94	94	41
UK	191	191	191	99	232	231	232	191	191	116
Ukraine	103	103	103	33	142	141	142	103	103	37
Turkey	103	103	103	9	114	113	116	103	103	12
Spain	191	191	191	82	227	227	229	190	190	94
South Korea	5	5	5	2	11	11	11	5	5	2
Saudi Arabia	34	34	34	6	49	48	49	34	34	11
Russia	159	159	159	24	163	164	164	159	159	26
Netherlands	132	132	132	134	194	192	194	132	132	147
Japan	63	63	63	32	69	70	70	63	63	35
Italy	331	331	331	128	355	352	355	331	331	143
Indonesia	69	69	69	11	85	85	85	69	69	14
Greece	101	101	101	60	171	171	171	101	101	68
Germany	189	189	189	114	223	224	225	189	189	127
Canada	166	166	166	52	183	183	184	166	166	63
Brazil	166	166	166	59	180	179	181	166	166	65
Australia	151	151	151	58	161	160	161	151	151	69
Argentina	159	159	159	43	189	188	190	159	159	47
Total	2407	2407	2407	980	2857	2848	2868	2406	2406	1117

**Table 4:** Demographics of longitudinal sample.

	*N*	Age	Gender	Education	Religious
USA	116	18–24 = 1% 25–34 = 4% 35–44 = 15% 45–54 = 22% 55–64 = 30% 65–75 = 24% 75–85 = 5% 85 + = 0%	Female = 71% Male = 30% Other = 0%	Primary education = 2% General secondary education = 22% Vocational education = 9% Higher education = 33% Bachelor’s degree = 21% Master’s degree = 11% PhD degree = 3%	No = 43% Yes = 57%
UK	241	18–24 = 1% 25–34 = 5% 35–44 = 14% 45–54 = 21% 55–64 = 21% 65–75 = 33% 75–85 = 5% 85 + = 1%	Female = 51% Male = 49% Other = 0%	Primary education = 0% General secondary education = 33% Vocational education = 24% Higher education = 14% Bachelor’s degree = 22% Master’s degree = 6% PhD degree = 0%	No = 68% Yes = 32%
Ukraine	154	18–24 = 4% 25–34 = 21% 35–44 = 11% 45–54 = 13% 55–64 = 38% 65–75 = 13% 75–85 = 0% 85 + = 0%	Female = 54% Male = 46% Other = 0%	Primary education = 0% General secondary education = 6% Vocational education = 15% Higher education = 45% Bachelor’s degree = 7% Master’s degree = 24% PhD degree = 3%	No = 38% Yes = 62%
Turkey	121	18–24 = 3% 25–34 = 26% 35–44 = 27% 45–54 = 17% 55–64 = 22% 65–75 = 4% 75–85 = 0% 85 + = 0%	Female = 56% Male = 44% Other = 0%	Primary education = 1% General secondary education = 1% Vocational education = 19% Higher education = 10% Bachelor’s degree = 56% Master’s degree = 10% PhD degree = 3%	No = 34% Yes = 66%
Spain	242	18–24 = 2% 25–34 = 6% 35–44 = 7% 45–54 = 19% 55–64 = 30% 65–75 = 32% 75–85 = 4% 85 + = 0%	Female = 47% Male = 53% Other = 0%	Primary education = 4% General secondary education = 19% Vocational education = 14% Higher education = 21% Bachelor’s degree = 30% Master’s degree = 7% PhD degree = 5%	No = 57% Yes = 43%
South Korea	12	18–24 = 0% 25–34 = 25% 35–44 = 42% 45–54 = 17% 55–64 = 17% 65–75 = 0% 75–85 = 0% 85 + = 0%	Female = 50% Male = 50% Other = 0%	Primary education = 0% General secondary education = 8% Vocational education = 0% Higher education = 25% Bachelor’s degree = 50% Master’s degree = 8% PhD degree = 8%	No = 50% Yes = 50%
Saudi Arabia	54	18–24 = 6% 25–34 = 37% 35–44 = 35% 45–54 = 13% 55–64 = 9% 65–75 = 0% 75–85 = 0% 85 + = 0%	Female = 52% Male = 48% Other = 0%	Primary education = 0% General secondary education = 11% Vocational education = 2% Higher education = 4% Bachelor’s degree = 74% Master’s degree = 7% PhD degree = 2%	No = 17% Yes = 83%
Russia	166	18–24 = 1% 25–34 = 10% 35–44 = 16% 45–54 = 26% 55–64 = 27% 65–75 = 20% 75–85 = 1% 85 + = 0%	Female = 58% Male = 42% Other = 0%	Primary education = 0% General secondary education = 5% Vocational education = 26% Higher education = 44% Bachelor’s degree = 9% Master’s degree = 13% PhD degree = 2%	No = 36% Yes = 64%
Netherlands	224	18–24 = 0% 25–34 = 0% 35–44 = 8% 45–54 = 19% 55–64 = 33% 65–75 = 30% 75–85 = 9% 85 + = 0%	Female = 55% Male = 45% Other = 0%	Primary education = 4% General secondary education = 25% Vocational education = 40% Higher education = 22% Bachelor’s degree = 4% Master’s degree = 5% PhD degree = 1%	No = 54% Yes = 46%
Japan	74	18–24 = 11% 25–34 = 15% 35–44 = 7% 45–54 = 16% 55–64 = 23% 65–75 = 23% 75–85 = 4% 85 + = 1%	Female = 53% Male = 47% Other = 0%	Primary education = 0% General secondary education = 12% Vocational education = 3% Higher education = 16% Bachelor’s degree = 62% Master’s degree = 5% PhD degree = 1%	No = 80% Yes = 20%
Italy	370	18–24 = 4% 25–34 = 10% 35–44 = 20% 45–54 = 18% 55–64 = 16% 65–75 = 30% 75–85 = 2% 85 + = 1%	Female = 46% Male = 54% Other = 0%	Primary education = 1% General secondary education = 9% Vocational education = 8% Higher education = 52% Bachelor’s degree = 6% Master’s degree = 21% PhD degree = 4%	No = 35% Yes = 65%
Indonesia	88	18–24 = 22% 25–34 = 41% 35–44 = 17% 45–54 = 12% 55–64 = 8% 65–75 = 0% 75–85 = 0% 85 + = 0%	Female = 42% Male = 58% Other = 0%	Primary education = 0% General secondary education = 28% Vocational education = 9% Higher education = 6% Bachelor’s degree = 53% Master’s degree = 3% PhD degree = 0%	No = 16% Yes = 84%
Greece	188	18–24 = 4% 25–34 = 5% 35–44 = 9% 45–54 = 20% 55–64 = 41% 65–75 = 20% 75–85 = 1% 85 + = 0%	Female = 47% Male = 53% Other = 0%	Primary education = 1% General secondary education = 3% Vocational education = 6% Higher education = 27% Bachelor’s degree = 45% Master’s degree = 15% PhD degree = 4%	No = 28% Yes = 72%
Germany	243	18–24 = 2% 25–34 = 3% 35–44 = 10% 45–54 = 19% 55–64 = 29% 65–75 = 34% 75–85 = 4% 85 + = 0%	Female = 52% Male = 48% Other = 0%	Primary education = 0% General secondary education = 7% Vocational education = 54% Higher education = 14% Bachelor’s degree = 9% Master’s degree = 13% PhD degree = 2%	No = 72% Yes = 28%
Canada	189	18–24 = 2% 25–34 = 6% 35–44 = 14% 45–54 = 30% 55–64 = 18% 65–75 = 25% 75–85 = 4% 85 + = 0%	Female = 50% Male = 50% Other = 0%	Primary education = 1% General secondary education = 22% Vocational education = 20% Higher education = 17% Bachelor’s degree = 26% Master’s degree = 12% PhD degree = 2%	No = 65% Yes = 35%
Brazil	190	18–24 = 6% 25–34 = 18% 35–44 = 17% 45–54 = 24% 55–64 = 19% 65–75 = 13% 75–85 = 2% 85 + = 0%	Female = 55% Male = 45% Other = 0%	Primary education = 1% General secondary education = 21% Vocational education = 14% Higher education = 26% Bachelor’s degree = 28% Master’s degree = 7% PhD degree = 2%	No = 26% Yes = 74%
Australia	172	18–24 = 0% 25–34 = 6% 35–44 = 16% 45–54 = 17% 55–64 = 24% 65–75 = 30% 75–85 = 7% 85 + = 1%	Female = 55% Male = 45% Other = 0%	Primary education = 1% General secondary education = 24% Vocational education = 24% Higher education = 17% Bachelor’s degree = 26% Master’s degree = 6% PhD degree = 1%	No = 63% Yes = 37%
Argentina	196	18–24 = 2% 25–34 = 15% 35–44 = 12% 45–54 = 24% 55–64 = 33% 65–75 = 12% 75–85 = 3% 85 + = 0%	Female = 62% Male = 38% Other = 0%	Primary education = 3% General secondary education = 24% Vocational education = 16% Higher education = 23% Bachelor’s degree = 26% Master’s degree = 8% PhD degree = 1%	No = 43% Yes = 57%

## Measures

### Predictor variables

#### Personal risk perception (first-order effects)

##### Risk perception infection

We asked: “How likely is it that the following will happen to you in the next few months? You will get infected with coronavirus” (1 = *Exceptionally unlikely*; 8 = *Already happened*).

##### Risk perception economic

We also ask how likely they thought their personal situation will get worse due to economic consequences of coronavirus (1 = *Exceptionally unlikely*; 8 = *Already happened*).

#### Subjective social and community norms (second-order effects)

##### Social norms

Participants indicated their agreement with: “Right now, people in my area should self-isolate and engage in social distancing” (-3 = *Strongly disagree*, 3 = *Strongly agree*).

#### Community punishment

“To what extent is your community punishing people who deviate from the rules that have been put in place in response to the coronavirus?” (1 = *Not at all*; 6 = *very much*).

#### Political and governmental perceptions (third-order effects)

##### Perceived government’s efficacy to fight COVID-19

We asked participants how much they trusted the government of their country to take the right measures to deal with the coronavirus pandemic (1 = *Not at all*; 5 = *A great deal*).

##### Clarity of communication

We measured the extent to which participants believe they are “getting clear, unambiguous messages about what to do about the coronavirus?” (1 = *messages are completely unclear/ambiguous*, 6 = *messages are very clear/unambiguous*).

##### Generic conspiracy beliefs

We used three items to assess participants’ perceptions of societal-level conspiracies (e.g., “I think that government agencies closely monitor all citizens; I think that politicians usually do not tell us the true motives for their decisions” (0 = *Certainly not 0%*, 10 = *Certainly 100%*).

##### Cultural tightness

We used country-level scores for a country’s position on the looseness-tightness spectrum. Additionally, we asked people to indicate to what extent they thought that the country they currently live in should have the following characteristics right now? (1 = *Have flexible social norms*, 9 = *Have rigid social norms*; 1 = *Be loose*, 9 = *Be tight*; 1 = *Treat people who don’t conform to norms kindly*, 9 = *Treat people who don’t conform to norms harsh*).

### Outcome variables

#### Health behavior

Respondents were asked how much they agreed with: “To minimize my chances of getting coronavirus, I” “…wash my hands more often”, “…avoid crowded spaces”, and “…put myself in quarantine” (-3 = *Strongly disagree*, 3 = *Strongly agree*). We assessed face mask use: "In the past week, I have covered my face in public places." (1 = *(Almost) never*, 2 = (*(Almost)* always).^1^ In addition, we asked: “In the past 7 days, how many days did you have in-person (face-to-face) contact with other people in general [friends or relatives]” (0 = *0 days*, 7 = *7 days*). We also inquired: “In the past week, how often did you leave your home? (1 = *I did not leave my home*, 4 = *Four times or more*).

#### Attitudes toward behavioral mandates

Participants indicated their agreement with: "I would sign a petition that supports…mandatory vaccination once a vaccine has been developed for coronavirus…mandatory quarantine for those that have coronavirus and those that have been exposed to the virus” (-3 = *Strongly disagree*, 3 = *Strongly agree*), and “I would join a protest against social distancing measures” (-2 = *Strongly disagree*, 2 = *Strongly agree*).

Also, we measured age, gender (0 = female; 1 = male), employment status, education, political view, religion, the date the survey was taken, as well as whether participants knew of any COVID-19 cases among friends and family.

### Strategy of analyses

We conducted our analyses in R (https://www.R-project.org/), using the workflow for open reproducible code in science (WORCS) to make the analyses reproducible ^39^. All code is available on GitHub. Mplus 8.0 was used to estimate the multilevel models with full information maximum likelihood estimation, which is robust to non-normality of residuals and makes use of all available data without imputing missing values. We automated analyses and tabulated results using the MplusAutomation (https://CRAN.R-project.org/package=MplusAutomation) and tidySEM R-packages (www.github.com/cjvanlissa/tidySEM).

To examine over-time predictive effects, we restructured the data to long format, with one observation per time point per participant. Table 5 shows how many time points were available for each variable. For each time point *t*, the dependent variable was taken at *t* + *1*. An autoregressive effect was included for the dependent variable, thus controlling for stability, and we included the time difference *Dt* as a control variable. The results should thus be interpreted in terms of change in the dependent variable. We present the most parsimonious model with only the direct effects, as preliminary tests of interactions between all predictors and *Dt* indicated convergence issues and few were reliable. Regarding predictor variables, we selected all available data that coincided with the available waves of the dependent variable. We entered the predictors simultaneously to isolate their unique effects, above and beyond any explanatory variance shared among predictors. When repeated measures were available for a predictor variable, it was used as a within-participants factor. Thus, for predictors with repeated measures, the predictor at each time point *t* was used to predict the dependent variable at *t* + *1*. If only a single assessment of a predictor variable was available, it was used as a between-participants factor.

**Table 5:** Observations per time point for health behaviors and attitudes toward behavioral mandates.

Wave	Health behaviors							Attitudes toward behavioral mandates		
	Hand washing	Avoiding crowds	Self-isolation/quarantine	Wearing face mask	Face-to-face contact with friends and family	Face to face contact with other people	Days per week people left their house	Mandatory vaccination	Mandatory quarantine	Protest containment measures
Baseline	2403	2404	2404	–	2391	2381	2404	2403	2403	–
1	–	–	–	–	–	–	–	–	–	–
2	–	–	–	–	–	–	–	–	–	–
3	–	–	–	–	–	–	–	–	–	–
4	933	933	932	–	–	–	–	1127	1127	–
5	–	–	–	–	1297	1288	1300	–	–	–
6	–	–	–	169	–	–	–	–	–	203
7	–	–	–	–	–	–	–	–	–	–
8	–	–	–	930	–	–	–	–	–	1066
9	–	–	–	–	–	–	–	–	–	–
10	–	–	–	–	–	–	–	–	–	–
11	–	–	–	–	–	–	–	–	–	–

### Open practices statement

The study was not formally preregistered but all data will be made available online upon publication. Full methodological details, including exact dates of the waves and questionnaires in all languages, are provided in the survey codebook (https://osf.io/qhyue). We conducted our analyses in R, using the workflow for open reproducible code in science to make the analyses reproducible. All code is available on GitHub (https://github.com/cjvanlissa/schumpe).

### Ethics statement

The study was approved by the Ethics Committees of the University of Groningen (PSY-1920-S-0390) and New York University Abu Dhabi (HRPP-2020-42).
